# Novel sulfonated poly(ether ether ketone)/triphenylamine hybrid membrane for vanadium redox flow battery applications

**DOI:** 10.1039/c8ra09695c

**Published:** 2019-01-29

**Authors:** Yizhou Quan, Gang Wang, Anfeng Li, Xiaoyan Wei, Feng Li, Jie Zhang, Jinwei Chen, Ruilin Wang

**Affiliations:** College of Materials Science and Engineering, Sichuan University Chengdu 610065 China electrowg100@scu.edu.cn rlwang@scu.edu.cn +86 2885418018 +86 2885418018

## Abstract

A novel sulfonated poly(ether ether ketone)/triphenylamine hybrid membrane with various triphenylamine loadings (1%, 2% and 5%) has been successfully fabricated. Optimum triphenylamine loading was confirmed by exploring the physicochemical properties and morphology of different membranes. The hybrid membrane exhibited lower vanadium permeability than pristine SPEEK membranes due to the acid–base interaction between amine groups and sulfonated groups. Introduction of triphenylamine also improved the proton conductivity because the nitrogen atom of triphenylamine can be protonated and contribute to the proton transfer. As the result, the hybrid membrane demonstrated higher ion selectivity compared with SPEEK and Nafion115 membranes. The VRFB single cell with SPEEK/TPAM-1% membrane showed better performance compared to a Nafion115 membrane at the current density of 60 mA cm^−2^. The SPEEK/TPAM hybrid membrane has great potential for VRFB application.

## Abstract 2

The novel TPAM hybrid membrane exhibited both lower vanadium permeability and higher proton conductivity than pristine SPEEK membrane.

## Graphical abstract

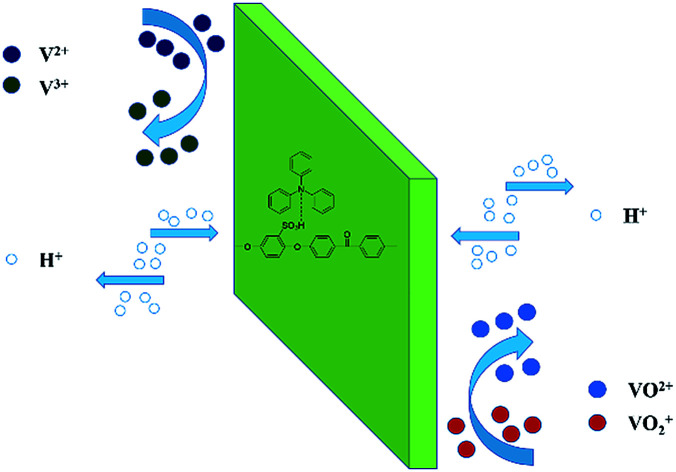

## 1. Introduction

As a large-scale energy storage technology, the redox flow battery (RFB) has unique characteristics, such as long cycle-life, high efficiency and environmental friendliness.^1–3^ Among various types of RFB technologies, the vanadium redox flow battery (VRFB) uses the same material in both half-cells, which avoids cross-contamination of the two half-cells' electrolytes and provides the electrolytes with potentially unlimited life. Therefore, it is especially suited for large stationary energy storage.^4^ VRFBs include two electrolyte tanks and a battery stack. V(ii)/V(iii) and V(iv)/V(v) couples in sulfuric acid solution are used as negative and positive electrolytes, respectively, which cyclically pump in the stacks.^5^

The proton exchange membrane (PEM) is a key part of a VRFB, it prevents positive and negative half-cells from cross-mixing, and meanwhile, it needs to transport charge-balancing ions, such as H^+^, SO_4_^2−^ and HSO_4_^−^.^6,7^ The properties of PEM determine the performance of VRFB, so that the ideal PEM is required to possess better chemical stability, high ion selectivity, low cost and excellent mechanical strength.^8,9^ The most commonly used commercial membrane is Dupont's Nafion® membrane, which exhibits high proton conductivity and outstanding chemical stability.^10^ However, high price and high vanadium permeability limit its further application.^11^ Therefore, the alternative PEM of VRFB needs to be explored.

To this day, fluorinated^12,13^ and non-perfluorinated membranes have been comprehensively developed, such as sulfonated poly(ether ether ketone) (SPEEK),^14–17^ sulfonated poly (diallyl-bisphenol ether ether ketone) (SDPEEK),^18^ sulfonated polyimide (SPI),^19^ polybenzimidazole (PBI)^20^ and sulfonated polysulfone (SPSF).^21^ Among these sulfonated aromatic polymers, SPEEK has been widely used in VRFB owing to its high ion selectivity, easy preparation and low cost.^22^ Nevertheless, the performance of SPEEK is greatly affected by the degree of sulfonation (DS).^23^ So researchers turn their attention to modify SPEEK to satisfy both high conductivity and low swelling ratio. In recent years, many methods of PEM modification have been reported, such as filling with inorganic particles like TiO_2_ and SiO_2_,^24–27^ blending polymers like PAN and PVDF,^28,29^ doping organic fillers^30,31^ and multilayer protection.^32,33^ Introducing the amine functional group to achieve acid–base blend membrane is the most frequently used strategy to modify the SPEEK membranes. Xi *et al.* coated the SPEEK with polydopamine to protect the membranes from being corroded by the strong acidic and oxidizing environment.^34^ Besides, Yan *et al.* used imidazolium-functionalized polysulfone as the base polymer and SPEEK as the acid polymer to prepare the amphoteric membrane for VRFB, which showed both higher coulombic efficiency and energy efficiency than Nafion212 membrane.^35^

The triphenylamine(TPAM) unit has been widely studied and applied in various optoelectronic materials and organic field effect transistors.^36^ It is a great electron donor unit^37^ and the nitrogen atom of TPAM can be protonated and functioned as an ion exchange site to improved proton conductivity.^38^ Furthermore, the acid–base interactions in the acid–base polymer blend membrane could decrease the swelling degree and the electrolyte corrosion, and improve the vanadium ions resistance and selectivity.^39^

Our group has been working on the membrane of VRFB for many years, such as the surface modification of Nafion membrane,^40^ the optimization and composite of SPEEK.^41,42^ Therefore, based on the above facts, we selected TPAM for modification and doping of SPEEK and prepared a novel SPEEK/TPAM hybrid membrane. The properties and corresponding VRFB cell performance of the hybrid membrane were investigated.

## 2. Experimental

### 2.1 Materials

Poly(ether ether ketone) (PEEK) (Victrex, PEEK 450PF) was dried under vacuum at 100 °C for 24 h. The Nafion115 membrane was purchased from DuPont Company, Nafion115 membrane was treated by 3wt% H_2_O_2_ solution at 80 °C for 1 h, deionized water at 80 °C for 30 min and 1 mol L^−1^ H_2_SO_4_ solution at 80 °C for 30 min. All the other reagents were provided by local chemical suppliers and used without further purification, including TPAM, *N*,*N*-dimethylacetamide (DMAC), H_2_SO_4_ (98 wt%), NaCl, NaOH, VOSO_4_ and MgSO_4_·7H_2_O.

### 2.2 Preparation of SPEEK/TPAM membrane

5 g PEEK was added into 100 mL H_2_SO_4_ (98 wt%) and the reactants were stirred at 45 °C for 4.5 h. Then the solution was poured into excess ice-cold water to terminate the sulfonated reaction. The SPEEK was washed until the pH reached neutrality, and dried under vacuum at 100 °C for 12 h. The DS of SPEEK was 55% determined by titration method.^43^ The SPEEK/TPAM membrane was made by the traditional solution casting method.^23^ 1.5 g SPEEK was dissolved into 10 mL DMAC and the TPAM was dissolved into the solution. The membrane was cast by pouring the solution on a stainless plate and dried at 80 °C for 10 h and 100 °C for 8 h. Then the membrane was soaked in 1 mol L^−1^ H_2_SO_4_ solution for 24 h, and stored in deionized water before use.

### 2.3 Membrane characterization

#### 2.3.1 Membrane morphology and thermogravimetric analysis

The surface and cross-section morphology of various membrane were analyzed by scanning electron microscope (FE-SEM, Hitachi S4800, Japan). X-ray photoelectron spectroscopy (XPS) was used to analyze the surface chemical composition of the S/TPAM membrane. Thermogravimetric analysis (TGA, NETZSCH STA 449 C) was used to investigate the thermal properties of SPEEK and SPEEK/TPAM membrane. The sample was heated at 20 °C min^−1^ from room temperature to 800 °C under argon atmosphere.

#### 2.3.2 Water uptake and swelling ratio

The weight and length of the wet membrane were quickly measured after wiping off excess water on the surface. After 24 h of drying in the vacuum oven at 100 °C, the weight and length of the membrane were measured. Water uptake (*W*_U_) and swelling ratio (*S*_R_) were evaluated according to the formulas (1) and (2):1
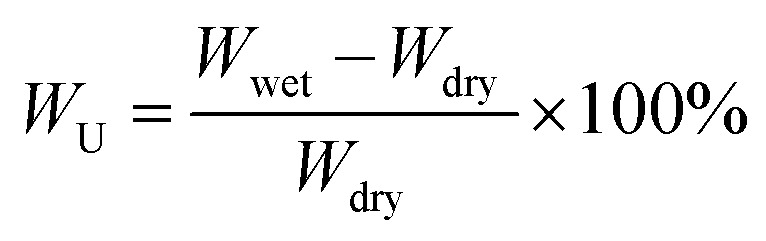
where *W*_wet_ and *W*_dry_ are the weights of wet and dry membranes, respectively.2
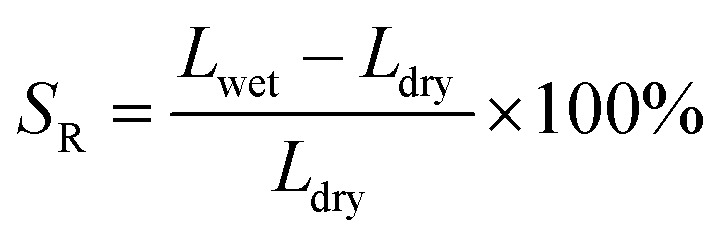
where *L*_wet_ and *L*_dry_ are the lengths of wet and dry membranes, respectively.

#### 2.3.3 Ion exchange capacity (IEC) and proton conductivity

IEC of the membrane was evaluated by the conventional titration method.^43^ The sample was soaked in 50 mL of 1 mol L^−1^ NaCl for 24 h, to make the Na^+^ and H^+^ exchange. The resultant solution was titrated with 0.01 mol L^−1^ NaOH, using phenolphthalein as indicator. IEC was evaluated according to the formula:3
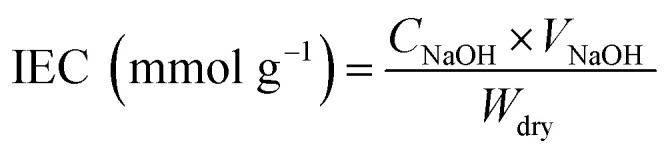
where *V*_NaOH_ is the volume of consumed NaOH solution and *C*_NaOH_ is the concentration of NaOH solution.

The proton conductivity of the membrane was measured by electrochemical impedance spectroscopy (EIS), using a Solartron 1287 + 1260 electrochemical station (USA, AMETEK, Inc.). The proton conductivity was evaluated according to the formula:4
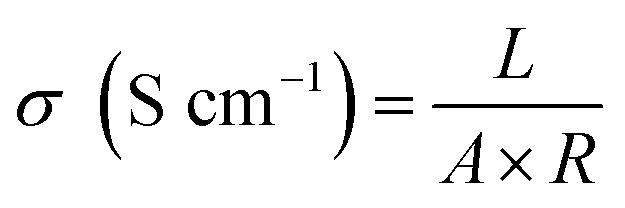
where *A* and *L* are the effective area and thickness of the sample membrane, and *R* is the sample membrane resistance.

#### 2.3.4 VO^2+^ permeability and selectivity

The method of VO^2+^ ion permeability test was described by the previous literature.^29^ The left reservoir was filled with 25 mL of 1 M VOSO_4_ (the concentration of the vanadium ions in the electrolyte were measured by 916 Ti-Touch (Metrohm) potentiometric titrator) in 3 M H_2_SO_4_ solution, and 25 mL of 1 M MgSO_4_ in 3 M H_2_SO_4_ solution was on the right to minimize the effect of osmotic pressure. The sample from the right side was taken out every 1 h to measure the concentration of VO^2+^ by a UV-vis spectrometer (TU-1900, China). The VO^2+^ permeability of the membrane was evaluated according to the formula:5
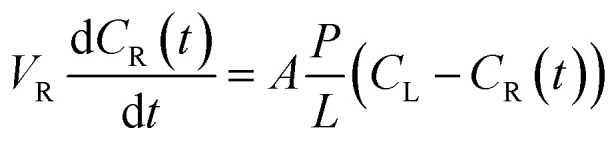
where *V*_R_ is the solution volume of the right reservoir, *C*_L_ and *C*_R_(*t*) are the VO^2+^ concentration in the left and right reservoirs respectively, *A* and *L* are the effective area and thickness of the sample membranes respectively, *P* is the VO^2+^ permeability.^44^

Ion selectivity (*S*) of the membrane was defined as the ratio of proton conductivity over VO^2+^ permeability, and it was evaluated according to the formula:6
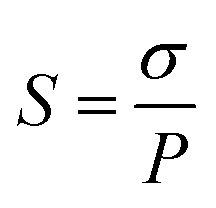


### 2.4 VRFB single cell test

The VRFB single cell was assembled by sandwiching a membrane with 5 cm × 5 cm effective area, using two pieces of 5 mm thickness graphite felt as electrodes, and two graphite polar plates and two copper foils as current collectors. 40 mL of 1.5 mol L^−1^ V^3.5+^ in 3 mol H_2_SO_4_ solution acted as the positive and negative electrolyte, respectively. Both of the electrolytes were cycled by the peristaltic pump (BT100L, LEAD FLUID, China) and the flow rate was set at 39 mL min^−1^. The cell was tested by a Land-CT2001A battery test system (Wuhan Land Co. Ltd.) with the current density of 60 mA cm^−2^, 1.65 V was the upper limit of charge voltage and 0.8 V was the lower limit of the charge voltage to avoid the corrosion of electrodes. The coulombic efficiency (CE), voltage efficiency (VE) and energy efficiency (EE) of the cell were calculated by formula (7)–(9):7
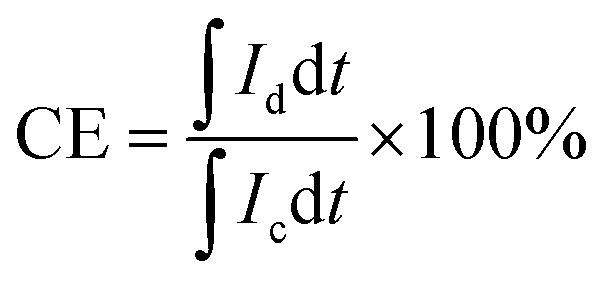
8
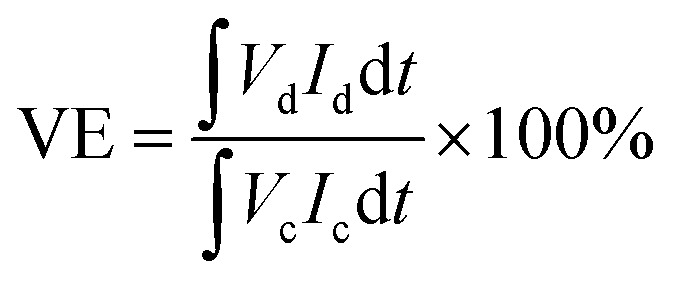
9
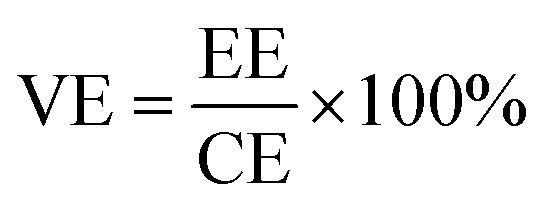
where *I*_c_ and *I*_d_ are the charge and discharge current, respectively; and *V*_c_ and *V*_d_ are the charge and discharge voltage, respectively.

### 2.5 Chemical stability

Chemical stability was detected by soaking the membranes in 30 mL of 1.5 mol L^−1^ VO_2_^+^ with 3 mol L^−1^ sulfuric acid for 30 days. And the weight loss of the membranes was calculated by formula (10).10
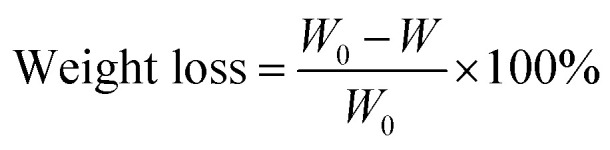
where *W*_0_ and *W* are the membrane weight before and after soaking into V(v) solution for 30 days.

## 3. Result and discussion

### 3.1 SEM and XPS characterizations

**Fig. 1:**
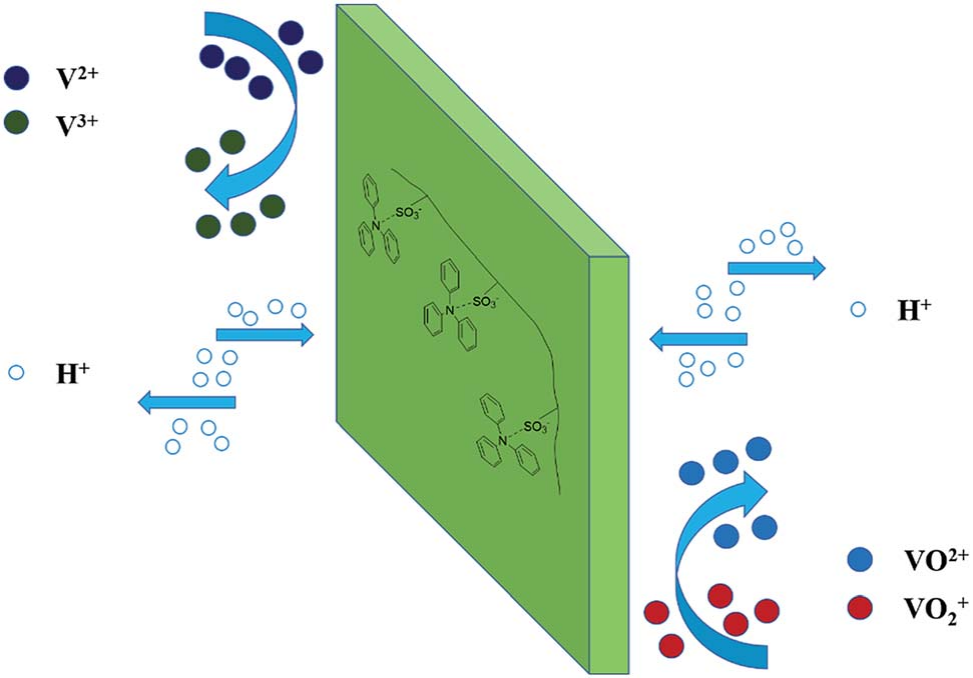
Proposed mechanism for proton transportation and vanadium ion permeation process in SPEEK/TPAM hybrid membrane.

SPEEK was firstly hybrid with the TPAM molecule to prepare the acid–base blend membrane. Fig. 1 illustrated a possible proton transportations mechanism for the SPEEK/TPAM hybrid membrane. The acid–base interaction of hydrogen bonding between TPAM and SPEEK built an effective proton channel, promoting the proton crossover the hybrid membrane. The membrane roughness from the wrinkles and grooves of TPAM block the transportation of vanadium ions.^45^

**Fig. 2:**
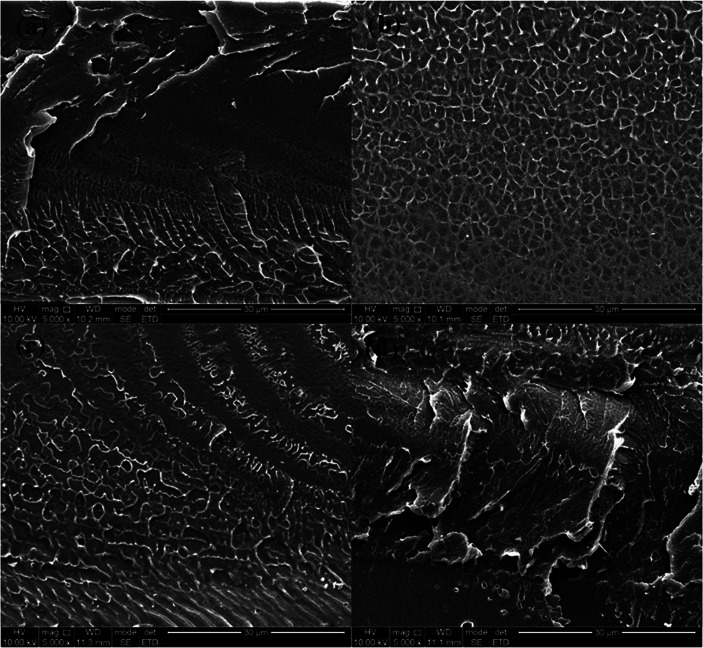
SEM images of the cross-section morphology of the SPEEK (a), S/TPAM-1% (b), S/TPAM-2% (c) and S/TPAM-5% (d).

**Fig. 3:**
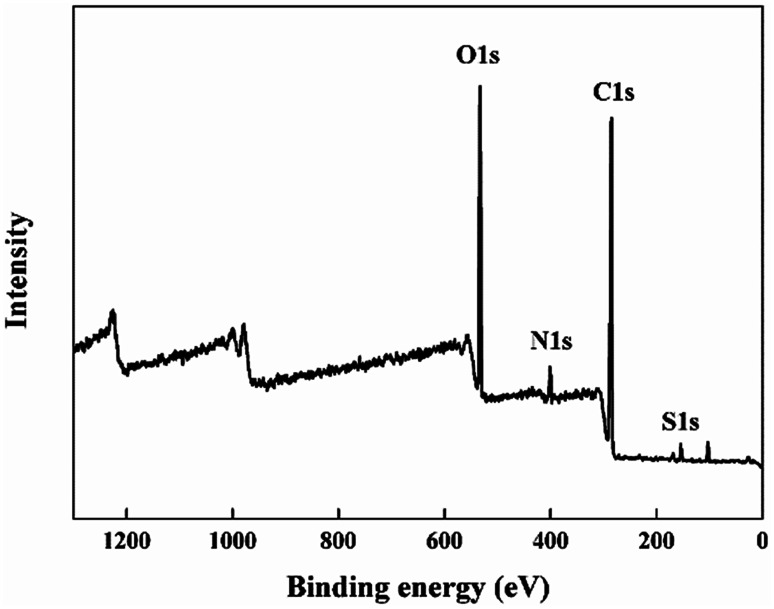
XPS spectra of the S/TPA-1% membrane.

Fig. 2(a–d) showed the cross-section morphology of pristine SPEEK membrane, SPEEK/TPAM (S/TPAM) membranes. In Fig. 2(a), the cross-section morphology of the pristine SPEEK membrane exhibited a smooth surface, while the hybrid membrane exhibited a relatively rough morphology in Fig. 2(b). The increased roughness is originated from the acid–base interaction.^46^ In Fig. 2(b), no cluster or aggregation appears, which meant that the TPAM molecule was well-distributed into the SPEEK matrix. However, the morphologies of S/TPAM-2% and S/TPAM-5% did not disperse as uniformly as S/TPAM-1%, some cluster and aggregation were observed in the membranes. XPS spectra of the S/TPAM-1% membrane was shown in Fig. 3. The peak of the N1s demonstrated that the TPAM was successfully doped into the SPEEK membrane.^47^

### 3.2 Physicochemical properties

The physicochemical properties of Nafion115, SPEEK membrane and S/TPAM membrane with different loadings were listed in Table 1. As shown in the list, the water uptake increased (*W*_U_), the swelling ratio (*S*_R_) and IEC decreased with the TPAM addition. This was possible attributed to the TPAM consumed the SO_3_H groups of SPEEK due to acid–base interaction.^48^

**Table 1:** The physicochemical property of Nafion115, SPEEK and S/TPAM membrane with 1%, 2% and 5% loading

Sample	Thickness (μm)	*W*_U_ (%)	*S*_R_ (%)	IEC (mmol g^−1^)	Proton conductivity (S cm^−1^)	VO^2+^ permeability (10^−7^ cm^2^ min^−1^)	Selectivity (10^4^ S min cm^−3^)
Nafion115	150	28.2	26.8	0.97	0.125	41.31	3.03
SPEEK	115	36.8	16.4	1.64	0.061	3.56	17.13
S/TPAM-1%	108	39.3	13.7	1.55	0.070	3.04	23.02
S/TPAM-2%	98	38.7	14.2	1.42	0.064	3.23	19.81
S/TPAM-5%	118	39.5	15.6	1.13	0.063	6.56	9.60

Moreover, because of the proton channel built by SPEEK and TPAM, the proton conductivity of the blend membrane was higher than the pristine membrane. However, the conductivity of membranes decreased with the increase of the TPAM content. It could be ascribed to some cluster and aggregation appearing in S/TPAM-2% and S/TPAM-5% influenced proton transportation in the SPEEK matrix.^49^

### 3.3 Vanadium permeability and selectivity

The vanadium permeability and selectivity were the significant properties of PEM applied in VRFB. PEM with high VO^2+^ permeability led to low coulombic efficiency, high rate of self-discharge and capacity reduction of VRFB.^50^

**Fig. 4:**
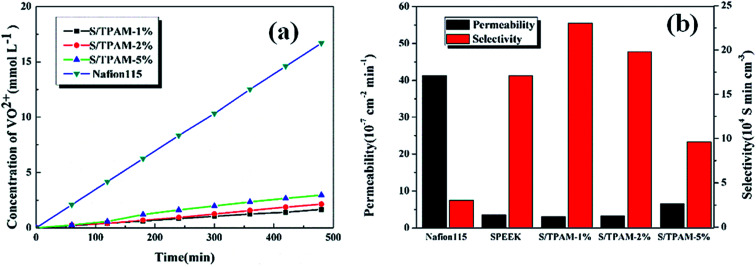
VO^2+^ permeability (a) and ion selectivity (b) of Nafion115 and S/TPAM membrane with different loading.

As shown in Fig. 4(a), S/TPAM blend membranes exhibited much lower VO^2+^ permeation than Nafion115 membrane. It was considered as the result of the lower SR of the blend membrane compared with Nafion115 membrane. The existence of ion clusters caused the larger water channel, which led to higher SR of Nafion115 membrane.^51^ Nevertheless, when increasing TPAM content, vanadium permeability of the hybrid membrane also increased significantly. It was because the cluster and aggregation appearing in S/TPAM-2% and S/TPAM-5% increased the water channel and led the transportation of VO^2+^.

Ion selectivity was a comprehensive factor to predict the performance of PEM applied in VRFB. As in Fig. 4(b), the S/TPAM-1% membrane presented the highest selectivity (2.33 × 10^5^ S min cm^−3^), which was much higher than the Nafion115 membrane (3.03 × 10^5^ S min cm^−3^). In summary, the S/TPAM-1% membrane possessed the best physicochemical properties among other membranes and it can be utilized in VRFB.

### 3.4 Thermogravimetric analysis

**Fig. 5:**
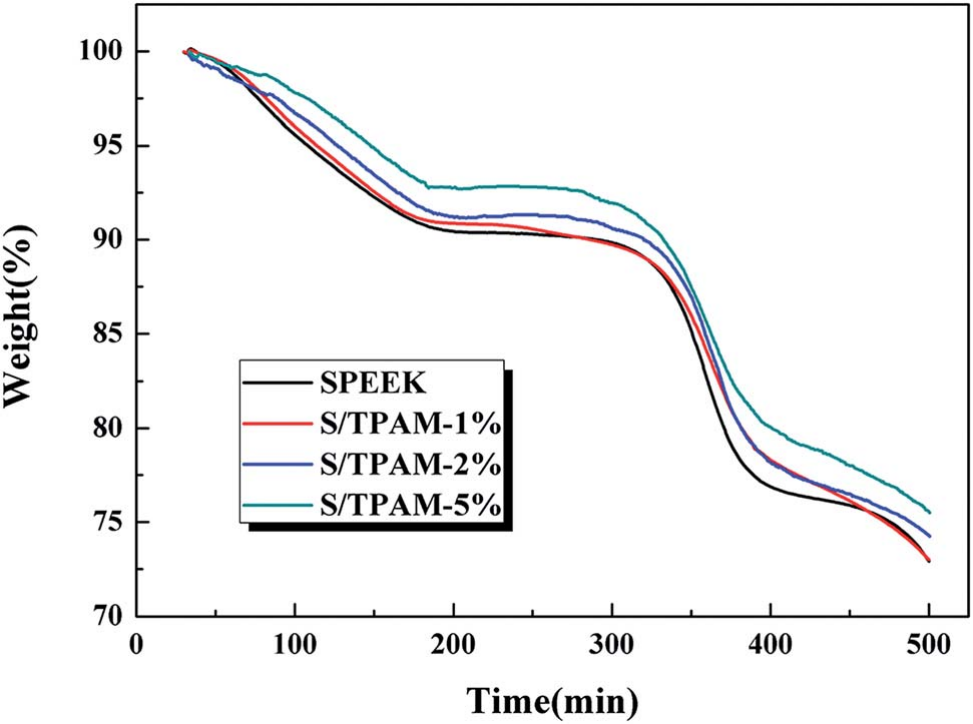
The TGA curves of SPEEK and S/TPAM-1%, S/TPAM-2% and S/TPAM-5%.

Fig. 5 displayed the TGA curves of SPEEK, S/TPAM-1%, S/TPAM-2% and S/TPAM-5%. All the membranes presented two steps of thermal degradation. The first mass lost at 320 °C was attributed to the degradation of sulfonated groups in SPEEK, while the second mass lost occurring at 470 °C was owing to the decomposition of SPEEK polymer backbones.^52^ We observed that the hybrid membranes exhibited lower mass loss than the mass loss of the pristine membrane from 320 °C to 370 °C, and the hybrid membranes showed better thermal stability with the increase of TPAM. Owing to the acid–base interaction between sulfonated groups and amine groups, a part of the sulfonated groups degraded at a higher temperature.^53^

### 3.5 Chemical stability

As listed in Table 2. Nafion115 showed the lowest weight loss owing to its molecular structure of perfluorosulfonic acid which exhibited the outstanding chemical stability. S/TPAM-1% membrane showed better stability than pristine SPEEK, it is attributed to the acid–base interactions between sulfonic groups of SPEEK and TPA. However, the weight loss of the hybrid membrane was still higher than that of Nafion115. The strategy to enhance the chemical stability of SPEEK needed to be further investigated.

**Table 2:** Chemical stability of Nafion115, SPEEK and S/TPAM-1%

Sample	Weight loss (%)
Nafion115	2.4
SPEEK	6.2
S/TPAM-1%	3.8

### 3.6 VRFB single cell test

**Fig. 6:**
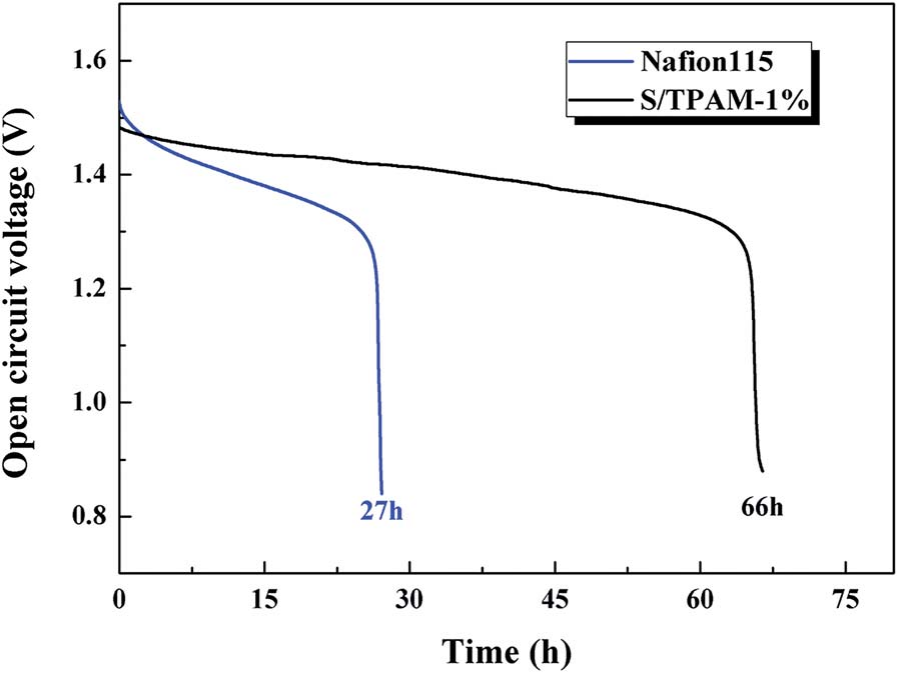
Self-discharge curves of the VRFB with Nafion115 and S/TPAM-1%.

Because of the excellent selectivity, S/TPAM-1% membrane was expected to be achieved in VRFB. Therefore, S/TPAM-1% membrane was utilized in the VRFB single cell test. The self-discharge curves of VRFB is illustrated in Fig. 6. The time of the open circuit voltage of VRFB with S/TPAM-1% decay to 0.8 V was 66 h, which is much longer than the self-discharge time of VRFB with Nafion115 (27 h). It is as the result of the vanadium permeability test.

**Fig. 7:**
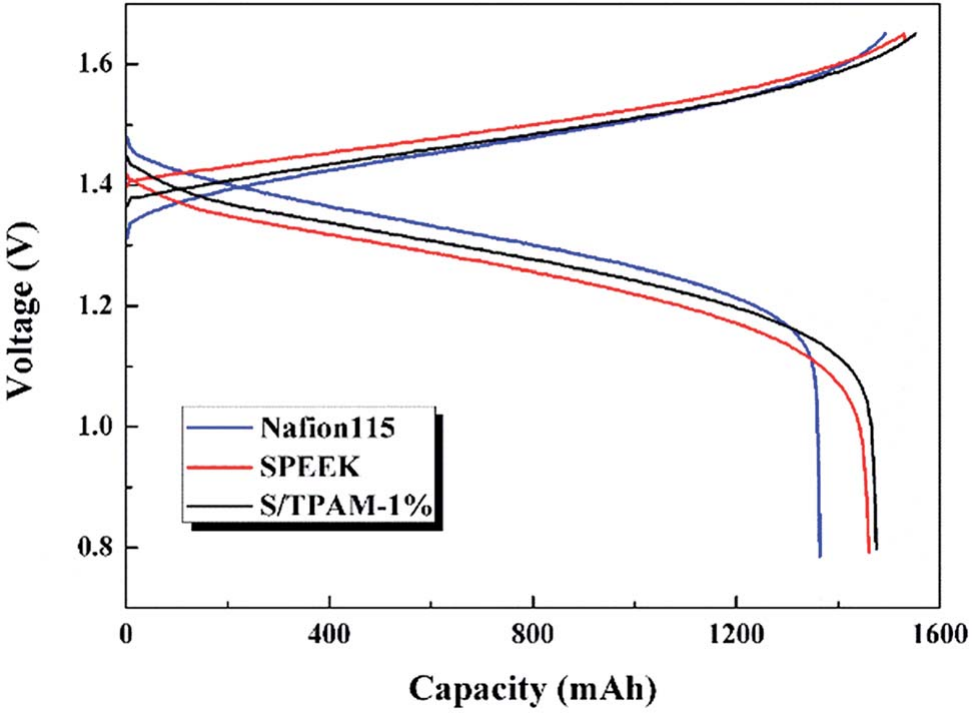
Charge–discharge curves of the cell with Nafion115, SPEEK and S/TPAM-1% at 60 mA cm^−2^.

Fig. 7 presented the charge–discharge curves of the cell with Nafion115, SPEEK and S/TPAM-1% membrane at 60 mA cm^−2^. Due to different proton conductivities of diverse membranes, the average charge voltage of VRFB had an order of SPEEK > S/TPAM > Nafion115, while the average discharge voltage of the cell had an order of Nafion115 > S/TPAM > SPEEK. Furthermore, S/TPAM-1% demonstrated higher charge capacity and higher discharge capacity than SPEEK and Nafion115 membrane, proving the superior balance between conductivity and vanadium permeability.

**Fig. 8:**
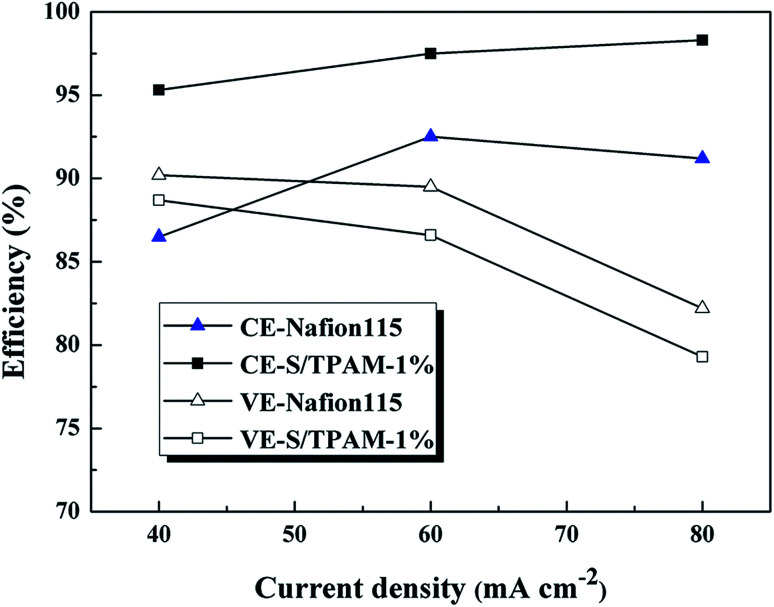
CE and VE of VRFB with Nafion115 and S/TPAM-1% membranes at constant current densities from 40 to 80 mA cm^−2^.

The efficiency of VRFB with Nafion115 and S/TPAM-1% membranes at constant current densities from 40 to 80 mA cm^−2^ were compared in Fig. 8. The CE of S/TPAM-1% membrane increased at the higher current density, while the CE of Nafion115 decreased at 80 mA cm^−2^. And the S/TPAM-1% demonstrated much higher CE than Nafion115 at the same current density. The VE of VRFB with both two membranes decreased at the higher current density. The S/TPAM-1% showed lower VE than Nafion115 at the same current density. The VRFB with S/TPAM-1% had the better rate performance than VRFB with Nafion115 at constant current densities from 40 to 80 mA cm^−2^.

**Fig. 9:**
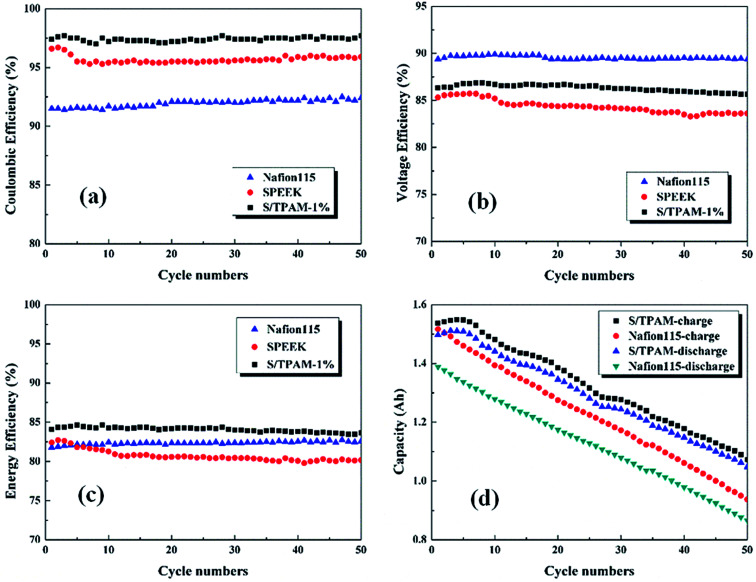
The cycling test of VRFB with Nafion115, SPEEK and S/TPAM-1% membrane at 60 mA cm^−2^.

The cycling performance of diverse membranes was explored at the current density of 60 mA cm^−2^, which was revealed in Fig. 9. As shown in Fig. 9(a), S/TPAM-1% had the highest CE up to 97.5% among the tested membranes. It was a result of the acid–base interaction between TPAM and sulfonated groups, and this interaction reduced the vanadium crossover. The lowest CE of Nafion115 (92.5%) membrane was caused by the large water channel and high swelling ratio. However, Nafion115 membrane possessed the higher VE (89.5%) than S/TPAM-1% (86.6%) and SPEEK (84.6%) in Fig. 9(b). In addition, the VE of SPEEK appeared a downward trend with the increase of cycle number, but it was not the case on Nafion115 and the hybrid membrane. The reason was that the introduction of TPAM into SPEEK improved the chemical stability of membranes. EE, served as an indicator of energy conversion in the charge–discharge cycle, as shown in Fig. 9(c). The EE of S/TPAM-1% (83.8%) was still higher than the Nafion115 (82.3%) and SPEEK (80.7%) membrane. It indicated the outstanding battery performance of the hybrid membrane, which was derived from a better balance between high conductivity and low vanadium cross-mixing. Moreover, the capacity reduction of the single cell was revealed in Fig. 9(d). Due to the great disparity of vanadium permeability, it was clear that the battery with S/TPAM-1% had the advantages of both higher charge–discharge capacity and slower capacity decay than those with Nafion115 membrane. These results demonstrated the excellent cycling stability of S/TPAM-1% membrane, that could promote the development of PEM for the long-life VRFB application.

## 4. Conclusion

A novel S/TPAM hybrid membrane was successfully prepared and first applied in VRFB. The introduction of TPAM can not only enhance the proton conductivity and chemical stability of SPEEK membrane with low DS but also, available, block the vanadium permeation. Furthermore, the higher CE, EE and charge–discharge capacity were obtained in VRFB single cell test with the S/TPAM-1% membrane, compared with commonly used commercial Nafion115 membrane, the low-cost and easy-prepared S/TPAM hybrid membrane could be a promising candidate for VRFB application.

## Conflicts of interest

There are no conflicts to declare.

## Supplementary Material
